# Dynamic Altered Amplitude of Low-Frequency Fluctuations in Patients With Major Depressive Disorder

**DOI:** 10.3389/fpsyt.2021.683610

**Published:** 2021-07-19

**Authors:** Ruiping Zheng, Yuan Chen, Yu Jiang, Mengmeng Wen, Bingqian Zhou, Shuying Li, Yarui Wei, Zhengui Yang, Caihong Wang, Jingliang Cheng, Yong Zhang, Shaoqiang Han

**Affiliations:** ^1^Department of Magnetic Resonance Imaging, The First Affiliated Hospital of Zhengzhou University, Zhengzhou, China; ^2^Department of Psychiatry, The First Affiliated Hospital of Zhengzhou University, Zhengzhou, China

**Keywords:** major depressive disorder, amplitude of low-frequency fluctuations, dynamics, intrinsic brain activity, resting-state fMRI

## Abstract

**Background:** Major depressive disorder (MDD) has demonstrated abnormalities of static intrinsic brain activity measured by amplitude of low-frequency fluctuation (ALFF). Recent studies regarding the resting-state functional magnetic resonance imaging (rs-fMRI) have found the brain activity is inherently dynamic over time. Little is known, however, regarding the temporal dynamics of local neural activity in MDD. Here, we investigated whether temporal dynamic changes in spontaneous neural activity are influenced by MDD.

**Methods:** We recruited 81 first-episode, drug-naive MDD patients and 64 age-, gender-, and education-matched healthy controls who underwent rs-fMRI. A sliding-window approach was then adopted for the estimation of dynamic ALFF (dALFF), which was used to measure time-varying brain activity and then compared between the two groups. The relationship between altered dALFF variability and clinical variables in MDD patients was also analyzed.

**Results:** MDD patients showed increased temporal variability (dALFF) mainly focused on the bilateral thalamus, the bilateral superior frontal gyrus, the right middle frontal gyrus, the bilateral cerebellum posterior lobe, and the vermis. Furthermore, increased dALFF variability values in the right thalamus and right cerebellum posterior lobe were positively correlated with MDD symptom severity.

**Conclusions:** The overall results suggest that altered temporal variability in corticocerebellar–thalamic–cortical circuit (CCTCC), involved in emotional, executive, and cognitive, is associated with drug-naive, first-episode MDD patients. Moreover, our study highlights the vital role of abnormal dynamic brain activity in the cerebellar hemisphere associated with CCTCC in MDD patients. These findings may provide novel insights into the pathophysiological mechanisms of MDD.

## Introduction

Major depressive disorder (MDD) is a common psychiatric illness, with clinical manifestations of persistently depressed mood, loss of interest, low self-esteem and energy, weight change, and cognitive dysfunction (1, 2). These heterogeneous symptoms often impair daily life function and raise the risk of suicide (3, 4). It is predicted that MDD may become the leading cause of disability in high-income countries by 2030 (5). Considering the high disability, high recurrence, severe distress, and heavy financial burden, it is important to achieve a deeper understanding of the underlying neural mechanism to reduce the risks posed by MDD.

To determine the neurobiological mechanisms of MDD, neuroimaging researches have attempted to discover the underlying mechanisms of MDD by investigating functional abnormalities in the brain of MDD patients. For example, an increasing number of abnormal resting-state networks have been found in MDD patients, such as hyperactivity in task performance and hyperconnectivity of default mode network and decreased connectivity within salience network (6–8), supporting internally directed and self-referential thought and involved in pathological rumination in MDD (9). Another finding is that the aberrance of limbic regions led to dysregulation of emotion in MDD (10–12). In addition, structural impairments including reduced gray-matter volume in frontal cortex, hippocampus, cingulate cortex, amygdala, thalamus, putamen, and striatum were observed in MDD patients (13–16). Furthermore, some researches focused on the abnormal local brain activity in MDD using resting-state functional magnetic resonance imaging (rs-fMRI). An rs-fMRI study reported that increased amplitude of low-frequency fluctuation (ALFF) in the right ventral median frontal gyrus and higher ALFF in the right putamen in MDD patients (17). Another article showed increased ALFF primarily in the forebrain, while there was decreased ALFF in the posterior brain regions in treatment-naive MDD patients (18). A meta-analysis found decreased ALFF activity of the cerebellum in drug-naive MDD patients and increased ALFF activity in the anterior cingulate cortex (19). Liu et al. used fractional ALFF (fALFF), an improved ALFF method to measure the ratio of power spectrum of low-frequency range to that of the whole frequency range. In addition, they found that patients with MDD showed significantly decreased fALFF in the right cerebellum posterior lobe (CPL), left parahippocampal gyrus, and right middle frontal gyrus (MFG) and increased fALFF in the left superior occipital gyrus/cuneus (20). However, most of these studies were based on the implicit assumption that brain activity remains stationary during rs-fMRI scanning. Recently, an accumulating number of researches suggest that brain activity is dynamic over time (21–27).

Dynamic brain activity may indicate information on the variability in the strength or spatial dynamic organization of the brain, according to neuroimaging studies (28, 29). The fact that dynamics can capture uncontrollable yet repeated patterns of brain networks, which cannot be identified by static analysis, is a major inspiration for such studies (30, 31). As previously reported, dynamic functional connectivity can be used to distinguish patients from healthy controls in MDD, epilepsy, and schizophrenia (32–34). Xue et al. showed reduced dynamic regional homogeneity (dReHo) in both fusiform gyri and in the right temporal pole and hippocampus in MDD compared with healthy controls and further demonstrated the expression profiles of 16 gene modules were correlated with dReHo alterations in MDD (35). However, research into the dynamics of local brain activity is still limited. The traditional static ALFF (sALFF), as a novel, non-invasive method, can characterize the energy intensity of brain activity over a period of time and effectively delineate the potential pathophysiological mechanisms of diseases (29, 36, 37). ALFF is demonstrated to have higher test–retest reliability than ReHo and high intrascanner reliability and can provide reliable information for studies (38). By integrating the ALFF with the “sliding-window” method, the dynamic ALFF (dALFF), based on the theory that resting-state brain is a highly dynamic system on a variety of time scales, offers a new way to evaluate the variance of ALFF over time by calculating the temporal variability of local brain activity amplitude among voxels (21, 22, 24, 28, 39–43). Put in a nutshell, the sALFF demonstrates the stable activity intensity of brain regions, which represents baseline energy consumption for sustaining essential brain functions. The dALFF reflects the plasticity and flexibility of spontaneous brain activity through the variability of energy expenditure. Taken together, we combine sALFF and dALFF to explore the abnormality of brain activity in MDD patients, which may provide novel insight in the study of MDD.

Here, we employed the ALFF combined with a sliding-window approach to assess the temporal variability of intrinsic brain activity in MDD patients. We expected that patients with MDD would show altered dALFF patterns compared to those of HCs. Furthermore, dALFF may detect some underlying abnormal intrinsic brain activity that sALFF cannot obtain, which can deepen our understanding of the physiological mechanism of MDD. We also hypothesized that the dynamic indexes may be associated with MDD clinical characteristics.

## Methods

The institutional review board of the First Affiliated Hospital of Zhengzhou University approved this prospective study, and written informed consent was obtained from each subject.

### Participants

This study consists of 145 participants totally, including 81 first-episode drug-naive individuals with MDD (63% female) and 64 age-, gender-, and education level–matched healthy control subjects (50% female). The diagnosis of first-episode MDD was conducted according to the Structured Clinical Interview of the *Diagnostic and Statistical Manual of Mental Disorders, Fourth Edition* (*DSM-IV*) Patient Edition. The severity of MDD was assessed with the 24-item Hamilton Depression Scale (HAMD) (44, 45). All healthy control participants were screened for a current or past diagnosis of any Axis I or II disorder using the Structured Clinical Interview of the *DSM-IV* Non–Patient Edition and Structured Clinical Interview for *DSM-IV* Axis II Personality Disorders. Inclusion criteria of MDD included (1) 10–60 years of age and right-handed; (2) drug-naive and at first episode of depression; (3) currently suffering an episode of depression with HAMD total score ≥21; and (4) a duration of depression >2 weeks but ≤40 weeks. Exclusion criteria included (1) a history of medical condition of antipsychotic medicine; (2) other current or past psychiatric disorders such as bipolar or schizophrenia; (3) a history of cardiovascular or other serious systemic diseases; (4) dementia; (5) other neurological disease or prior head trauma leading to cognitive disorder; (6) current alcohol or substance addiction such as alcohol, tobacco, or drug dependence; (7) head movement >2.5 mm or 2.5°. All healthy participants were right-handed and had no first-degree family history of psychiatric disorders.

### MRI Data Acquisition

MR data acquisition was conducted on a 3-T GE MR scanner (Discovery 750 System, Milwaukee, WI, USA) with a 16-channel head coil. The rs-fMRI data were obtained using the following parameters: 32 axial slices without slice gap, repetition time (TR)/echo time = 2,000/41 ms, field of view = 220 × 220 mm^2^, in-plane matrix = 64 × 64, section thickness = 5 mm, slice gap = 0.4 mm, flip angle = 90°, and a total of 240 volumes and lasted 480 s for each subject. All participants were placed in a supine position with wearing earplugs to reduce the noise. To reduce head excessive motion, a foam pad was fixed on the both sides of head. During MR scanning, all participants were required to keep their eyes closed and heads still without falling asleep and try not to think about anything in particular.

### Data Preprocessing

Functional images were preprocessed using a software (Data Processing and Analysis for Brain Imaging toolbox; http://rfmri.org/DPABI) (46). The main steps were included: (1) exclusion of the initial 10 volumes to ensure signal stability; (2) slice timing and realignment; (3) spatial normalization to the standard Montreal Neurological Institute (MNI 152) space and resampled to 3 × 3 × 3 mm^3^; (4) spatial smoothing using a 6-mm full-width half-maximum Gaussian kernel; (5) detrending the BOLD signals to correct a linear trend; (6) regression out of the nuisance covariates including the averaged signals from global mean signals (22, 28, 30, 47), cerebrospinal fluid signals, white matter signals, and Friston-24 head motion parameters; (7) temporal filtering (bandpass, 0.01–0.08 Hz) of BOLD signals; (8) to exclude the influence of head motion and ensure the contiguous time points, scrubbing with cubic spline interpolation was used; (9) additionally, to evaluate the head movement, we also calculate the mean frame-wise displacement (FD) (48, 49). In the group-level analysis, we also used the mean FD as a covariate to reduce the impact of motion artifact in the fMRI signal.

### Calculation of dALFF

A sliding-window approach was applied to characterize the dynamic patterns equipped in Dynamic Brain Connectome (DynamicBC) toolbox (V2.2 http://restfmri.net/forum/DynamicBC) (42). Window length is an important parameter in resting-state dynamics computation. A shorter window length may increase the risk of introducing spurious fluctuations in the observed dALFF (50). By contrast, a longer window length may hinder the description of the temporal variability dynamics of ALFF (28). Based on this, according to the previous researches (22, 28, 51), we selected a window length of 50 TRs (100 s) and a window overlap of 60% (step size by 20 TRs) to compute the dALFF of each participant. The ALFF maps for each subject were computed within each window, generating a series of ALFF maps. Subsequently, the variance of dALFF maps across time was calculated to measure the temporal variability of intrinsic brain activity. Finally, the dALFF variability of all participants was then transformed into standardized *z* scores by subtracting the mean and dividing by the SD across each voxel to enhance data normality. To verify whether dALFF and sALFF exhibited similar or complementary information for our deep understanding of the neuropathological mechanisms about MDD, we obtained the sALFF map of each participant and then transformed into standardized *z* scores (37).

### Statistical Analysis

Demographic and clinical results were evaluated using a set of independent two-sample *t*-tests, *p* < 0.05 was considered as statistically significant including age and education, and χ^2^-test was used for gender. In order to further investigate the variations in temporal variability of dALFF, a two-sample *t*-test was conducted between the MDD and HC groups, with age, gender, education level, and mean FD as covariates. Multiple comparison was corrected for two-sample *t-*test using a topological false discovery rate (FDR) approach, with the initial height threshold of uncorrected *p* < 0.001 and topological FDR: *q* < 0.05. Similarly, two-sample *t*-test with the same covariates was applied to assess group differences of sALFF.

### Clinical Correlation Analysis

Once significant differences in dALFF and sALFF were detected in any brain regions, we extracted the mean dALFF variability values of the region of interest (ROI) of each dALFF and mean sALFF values of ROI of each sALFF using toolkit (rs-fMRI data analysis, http://www.restfmri.net/forum/REST) (52). Then, a two-tailed partial correlation analysis was conducted to further assess the relationship between the mean values (dALFF variability and sALFF) and clinical variables (HAMD score) in the MDD group, controlling for age, gender, education level, and head motion. A statistically significant threshold of *p* < 0.05/8 (Bonferroni corrected) was set for all correlation analyses.

### Validation Analysis

We validated our main results with different window lengths and different overlap rates (30 TRs, 0.6 overlap; 80 TRs, 0.6 overlap; 50 TRs, 0.8 overlap). An ALFF map was obtained for each sliding window, and the dALFF of each voxel was standardized using *z* transformation.

To test the reproducibility of our main findings, we randomly selected 40 cases from 81 MDD patients as the MDD group compared with 64 in the HC group.

## Results

### Demographics and Clinical Characteristics

The demographics and clinical features of the two groups are summarized in Table 1. No significant differences were detected between MDD patients and HC subjects in terms of demographic characteristics, such as age, gender, and education level. For MDD patients, the HAMD scores ranged from 21 to 68.

**Table 1:** Demographic and clinical characteristics of the subjects.

**Variables**	**MDD (*n* = 81)**	**HC (*n* = 64)**	***t*/χ^**2**^**	***P*-value**
Age (years)	17.46 ± 6.13	16.02 ± 1.61	−0.709	0.410
Gender (male/female)	30/51	32/32	2.455	0.117
Education (years)	10.16 ± 2.70	10.83 ± 4.49	−1.049	0.297
HAMD-24 (score)	33.92 ± 9.04	NA	-	-

*MDD, major depressive disorder; HC, healthy control subjects; HAMD-24, 24-item Hamilton Depression Rating Scale; NA, not applicable*.

### dALFF Variability Results

**Figure 1:**
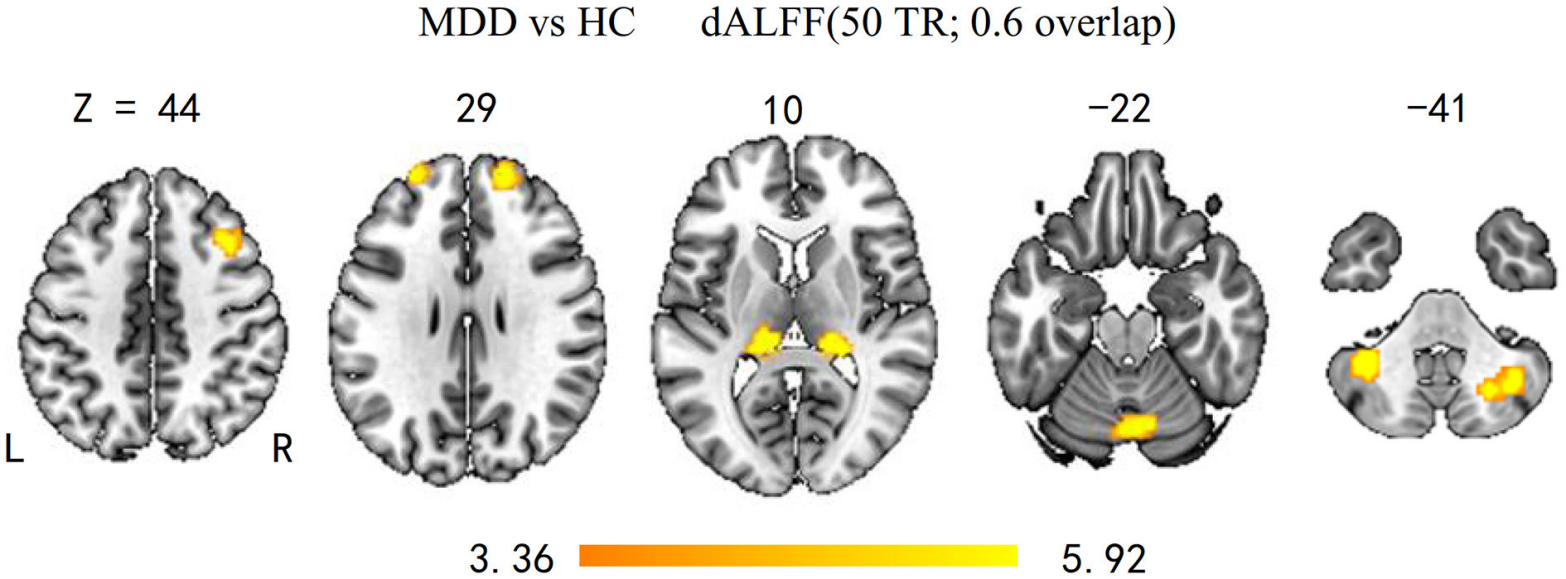
Brain regions with significant group differences in dALFF variability (50 TRs; 0.6 overlap). Group differences of temporal variability of dALFF between the MDD and HC groups were identified using a two-sample *t*-test. The statistical significance level was set at *p* < 0.05, topological FDR corrected. Patients with MDD showed significantly increased dALFF variability in the bilateral CPL, SFG, thalamus, and right MFG and vermis. dALFF, dynamic amplitude of low-frequency fluctuation; MDD, major depressive disorder; HC, healthy control; CPL, cerebellum posterior lobe; SFG, superior frontal gyrus; MFG, middle frontal gyrus.

As shown in Figure 1 and Table 2, the main results reported were based on the dALFF analysis using 50 TRs (100 s) as the window length. According to two-sample *t*-test, MDD patients, relative to controls, showed higher dALFF variability in the bilateral thalamus, bilateral superior frontal gyrus (SFG), bilateral CPL, right MFG, and vermis (*q* < 0.05, topological FDR corrected).

**Table 2:** Brain regions showing significant differences in dALFF between MDD patients and HC subjects.

**Regions**	**MNI coordinates**			**Voxels**	***T*-value**
	***x***	***Y***	***z***		
Thalamus_L	−12	−30	12	45	5.68
Thalamus_R	18	−33	12	57	5.40
Superior frontal gyrus_L	−27	57	30	24	5.16
Superior frontal gyrus_R	18	54	30	34	5.92
Middle frontal gyrus_R	39	18	42	29	5.22
Cerebellum posterior lobe_R	33	−63	−36	54	5.87
Cerebellum posterior lobe_L	−36	−51	−42	38	5.88
Vermis	6	−69	−18	46	4.88

*dALFF, dynamic amplitude of low-frequency fluctuation; MDD, major depressive disorder; HC, healthy control; MNI, Montreal Neurological Institute; L, left; R, right*.

Compared with HC, the MDD group showed decreased sALFF in the right CPL, left calcarine, left occipital, left fusiform gyrus, and right calcarine. The increased sALFF was located in the vermis (*q* < 0.05, topological FDR corrected) (Supplementary Figure 1 and Supplementary Table 1).

### Correlation Analysis Results

**Figure 2:**
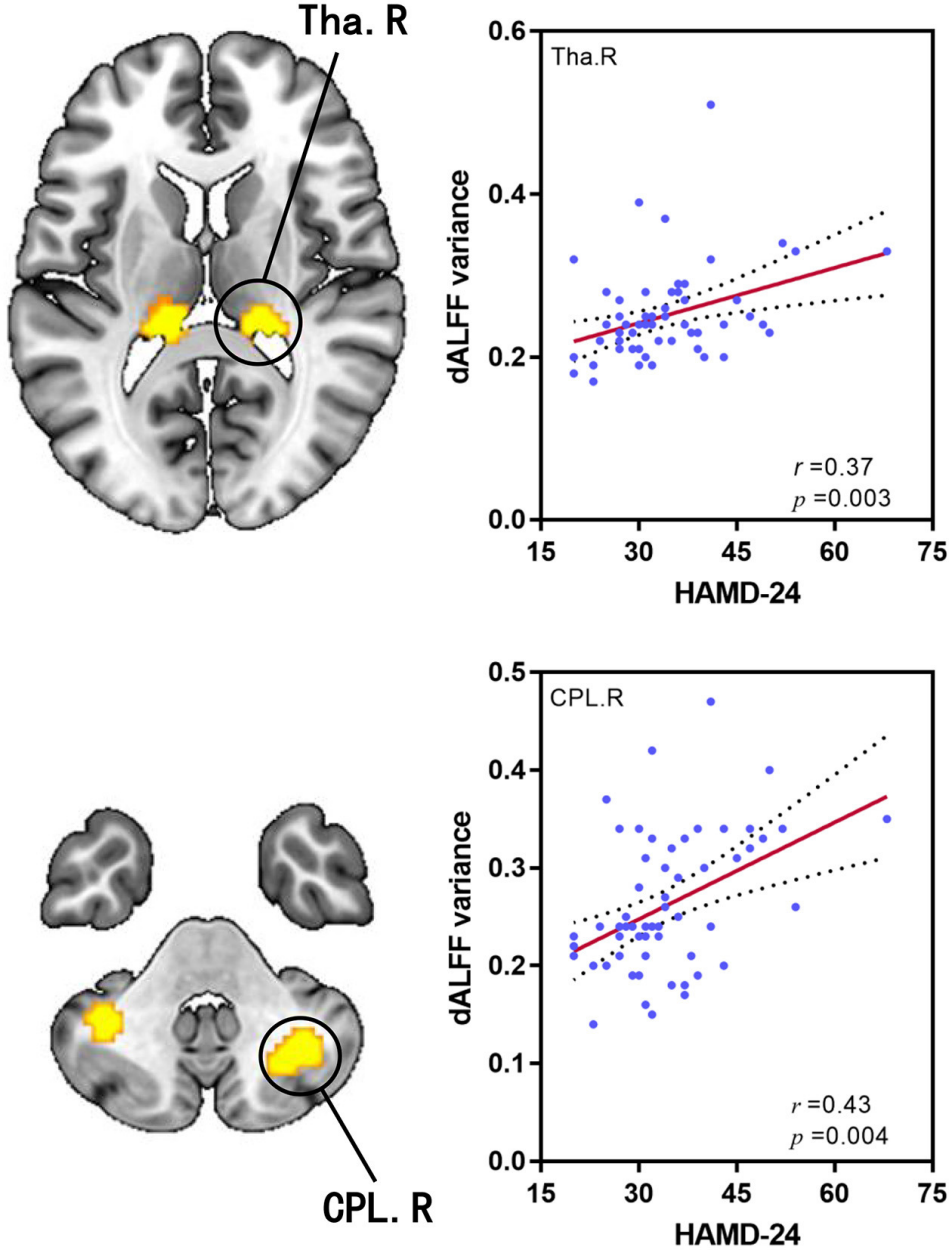
dALFF variability in the right thalamus and that in CPL were positively correlated with HAMD score of patients with MDD. dALFF, dynamic amplitude of low-frequency fluctuation; Tha, thalamus; CPL, cerebellum posterior lobe; HAMD, Hamilton Depression Rating Scale; MDD, major depressive disorder.

The 24-HAMD score was positively associated with dALFF variability in the right CPL and the right thalamus (*r* = 0.43, *p* = 0.004; *r* = 0.37, *p* = 0.003, Bonferroni corrected, respectively, Figure 2); No significant correlation was found in the relationship between abnormal sALFF and HAMD score in MDD patients.

### Validation Results

In our study, we validated our results by using different sliding-window lengths and different overlap rates. Finally, the results of the remaining two window lengths (30 TRs, 0.6 overlap; 80 TRs, 0.6 overlap) and one another overlap (50 TRs, 0.8 overlap) were presented in Supplementary Figures 2–4.

We randomly selected 40 cases from the total 84 patients as MDD group compared with 64 in the HC group, and this validation analysis replicated this higher dALFF variability of the corticocerebellar–thalamic–cortical circuit (CCTCC), and the detailed results are presented in Supplementary Figure 5.

## Discussion

To the best of our knowledge, we observed that only several researches employed a dALFF method to elucidate the dynamics of spontaneous neural-activity characteristics of MDD. In our study, increased dALFF variability in patients with MDD was prominently located in CCTCC, including bilateral thalamus, bilateral SFG, bilateral CPL, right MFG, and vermis, representing the anatomical substratum of functional networks governing behavior: executive, salience, default mode, dorsal attentional, and motor networks (53). Furthermore, the altered temporal ALFF variability values in right CPL and right thalamus had a significantly positive correlation in MDD patients. Overall, this study provides convincing evidence for dysfunction of intrinsic brain activity in CCTCC of MDD patients, which may underscore the importance of considering brain dynamics and truly inform the potential mechanism underlying MDD.

MDD showed increased dALFF in CCTCC, which are involved in emotional, executive, and cognitive functions. Of note, emotional dysfunction and cognitive deficits are the most prominent features of MDD (1), and Pessoa (54) suggested that emotion and cognition are often integrated and jointly contribute to behavior. Although previous neuroimaging studies have shown that CCTCC may be the primary abnormality in schizophrenia (55–58), the dysfunction of CCTCC has not been reported in MDD. CCTCC is the substrate of synchrony, which can integrate cerebral cortex and the cerebellum to ensure fluidly coordinating sequences of thought and action, occurring as a consequence of very rapid online processing and feedback (55). According to the hypothesis of cognitive disorder, the emergence of psychiatric illnesses may result from the altered of specific parts of the CCTCC, which could lead to the dysfunction of mental coordination processes, such as schizophrenia and Parkinson disease (55, 59–61). MDD is a complicated disease with the dysfunction of cortical and subcortical structures that modulate cognitive and emotional aspects of behavior and the deficiency of neural systems related to decision-making and energy–vitality in thought and action. Therefore, we speculated that increased dALFF variability in the CCTCC may deepen our sight on the abnormal cognitive and emotional processing in MDD patients.

Our study found increased dALFF in the bilateral CPL, whereas there was a decreased sALFF in the right CPL, showing dysfunctional intrinsic activity of the posterior cerebellum in MDD. The cerebellum has proven bidirectional connections with brain areas involved in emotional control and the interpretation of socially salient emotional information, according to anatomic proof (62). In particular, substantial associations have been established between the cerebellum and the posterior parietal and prefrontal cortical regions (63–66), as well as limbic regions, including the amygdala, the hippocampus, and the septal nuclei (67, 68). The cerebellum, as part of CCTCC, has been proposed to have an activity in the production of a variety of symptoms and higher cognitive impairments in mental disorders (69–72). Abnormal structure and function in the cerebellum have been demonstrated in MDD patients (73), including abnormalities of gray-matter volume (74, 75), glucose metabolism (76, 77), functional activity, and connectivity (78–84). A previous meta-analysis demonstrated decreased sALFF in the bilateral cerebellum in MDD (85), which was in line with our results. Moreover, the current findings of increased dALFF (more variability) in cerebellum were in line with previous studies and may reflect the instability of neurofluctuation and information integration in MDD patients. Taken together, we speculate that the increased dALFF in the bilateral CPL and decreased sALFF in the right CPL may reflect the abnormity or dysfunction of cerebellum, which may disrupt brain top-down processing in emotional, executive, and cognitive functions.

We found another considerably increased dALFF and sALFF in the vermis. The vermis, also known as the limbic cerebellum, has been reported to have wide connections with the limbic systems, thus providing the anatomic substrate for cerebellar involvement in emotional and affective behaviors (66, 68, 86, 87). Interestingly, several articles demonstrated that activation of the posterior cerebellar vermis has been identified when participants process their own traumatic encounter, whereas activation of the posterior cerebellar hemisphere has been found when subjects experience empathy for the suffering of others (88, 89). Above all, these results indicate that the vermis is essential to emotional/affective processing. In our study, the increased dALFF variability and sALFF exhibit aberrant temporal fluctuation and hyperactivation of local brain activity in these regions in MDD patients. Such abnormalities may disable to engage the network between limbic cerebellum and limbic cerebrum in emotion regulation in MDD.

We observed increased dALFF in the bilateral thalamus in MDD relative to the HC group, suggesting that abnormal brain instantaneous activity in thalamus may affect the association of CCTCC. The thalamus is an integral part of the emotional salience network, emotion modulation network, and cognitive/executive network (90), which are associated with MDD. The volume atrophy of thalamus was thought to better account for the deficits in top-down regulation of negative emotions among persons more vulnerable to developing depressive state (91). In addition, neuroimaging study has demonstrated that increased thalamic functional connectivity was related to decreased cognitive function in disease conditions (92–96). Notably, the present study also showed positive association between the dALFF variability in the right thalamus and the HAMD-24 score, suggesting that the dALFF variability of the right thalamus could progressively increase during the development of the severity of MDD. Therefore, these findings suggest that increased dALFF in the thalamus may disturb cognitive activity in MDD.

Our study found increased dALFF variance in the bilateral SFG and the right MFG in the MDD group. SFG and MFG are essential parts of dorsolateral prefrontal cortex (DLPFC), which is the core region of depressive episode and maintenance, involving cognitive control such as biased attention, negative self-referential schemas, and rumination (97). Beyond that, the DLPFC is a critical part of dorsal neural system implicated in regulating the parameters of affective states (98). According to previous reports, the changes in the volume or function of DLPFC regions have been considered to be the most common abnormal region in MDD. For example, previous articles have shown hypermetabolism and hyperactivity in the right DLPFC in MDD patients with application of different imaging techniques (positron emission tomography, fMRI, transcranial magnetic stimulation) (99–106). And also, some articles have proved that the altered volume of DLPFC correlates well with the activation during working memory updating and during conscious negative emotion processing in fMRI studies (107–109). Thus, our findings are compatible with these previous studies, demonstrating that increased dALFF variability may underlie the phenomenon of abnormal cognitive control and affective states.

sALFF and dALFF revealed a small overlap group differences in our study, including right CPL and vermis. In addition, decreased sALFF in the bilateral calcarine, left occipital lobe, and fusiform gyrus was also observed. Several studies have reported alterations along the visual pathway including occipital lobe and calcarine, as well as more downstream areas (e.g., fusiform gyrus) during working memory, attention, and visual categorization tasks in MDD (110–112). And also, there are some differences in spatial activation patterns in visual regions between healthy and depressed individuals (113–118). In addition, increased dALFFs in the right CPL and thalamus were positively correlated with HAMD score, whereas those associations were not detected in sALFF. These findings suggest that dALFF provides complementary information to probing pathological changes in MDD.

This study has some limitations. First, the optimal window size for capturing the dynamics of brain activity is still uncertain. In current study, we chose 50 TRs as the window length, which was proposed in previous articles (22, 28, 50). In our validation analysis, the main results of 50 TRs were close to the results of different sliding-window lengths and different overlap, showing that our findings of dALFF variability were relatively stable. Second, dALFF was used in current research to detect the temporal dynamics of local brain activity. However, further studies could also explore other parameters to characterize dynamic features precisely. Third, the small sample may affect the power of the statistical analysis. Future studies with a large sample size are needed to provide a comprehensive interpretation of the findings.

## Conclusion

In our study, the most parsimonious conclusion is that patients with MDD exhibited increased temporal variability of dALFF in regions mainly focused on CCTCC implicated in emotional, executive, and cognitive. More broadly, the dALFF abnormalities in the right thalamus and the right CPL were associated with the severity of illness, whereas sALFF could not detect this association. This study may emphasize the importance of applying the dynamic local brain activity in the pathophysiology underlying MDD and also demonstrated that dALFF could be a potential imaging biomarker for the diagnosis of MDD.

## Future Directions

Our current study provide evidence for intrinsic brain activity changes associated with MDD in CCTCC, which previous literatures rarely have detected in MDD. This may be due to an artifact of methodological constraints shared across the existing literature, and it also shows the sensitivity of dALFF in monitoring intrinsic brain activity. We look forward to future work that advances beyond the methodological constraints of the existing literature in order to make meaningful inroads in detecting the pathological mechanism of MDD. In addition, the present work also highlights the vital role of abnormal dynamic brain activity in the cerebellar hemisphere associated with CCTCC in MDD patients. Thus, future work could seek for a more comprehensive evidence to validate our speculation that the dysregulation of neuron in the cerebellar hemisphere may lead to the dysfunction of CCTCC in MDD.

## Data Availability Statement

The data analyzed in this study is subject to the following licenses/restrictions: The datasets used and analyzed during the current study are available from the corresponding author on reasonable request. Requests to access these datasets should be directed to fccchengjl@zzu.edu.cn.

## Ethics Statement

The studies involving human participants were reviewed and approved by Zhengzhou University First Affiliated Hospital. Written informed consent to participate in this study was provided by the participants' legal guardian/next of kin.

## Author Contributions

RZ, YC, MW, and SH conceived and designed the study. RZ and MW supervised the conduct of the study. YC, YJ, BZ, SL, and ZY are responsible for data acquisition. RZ and YC analyzed the data and takes responsibility for the paper. RZ, YC, YJ, and YW assisted with literature review. RZ, YC, and SH drafted the initial manuscript. JC, YZ, and SH reviewed and revised the manuscript. All authors read and approved the final manuscript.

## Conflict of Interest

The authors declare that the research was conducted in the absence of any commercial or financial relationships that could be construed as a potential conflict of interest.
