# Supplementary figures and images for: Dynamic Altered Amplitude of Low-Frequency Fluctuations in Patients With Major Depressive Disorder
Source: Front Psychiatry. 2021 Jul 19;12:683610. doi: 10.3389/fpsyt.2021.683610 (PMC8328277; doi:10.3389/fpsyt.2021.683610)

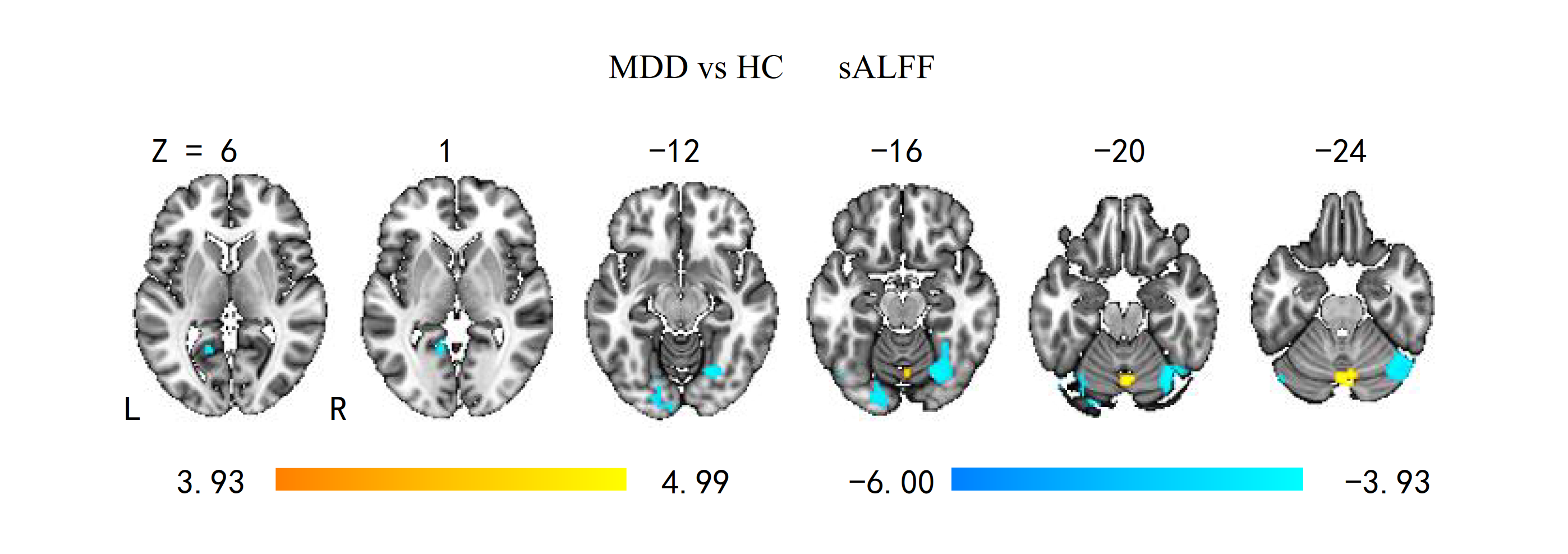

Supplement: Supplementary file 1 [file Image_1.TIF]

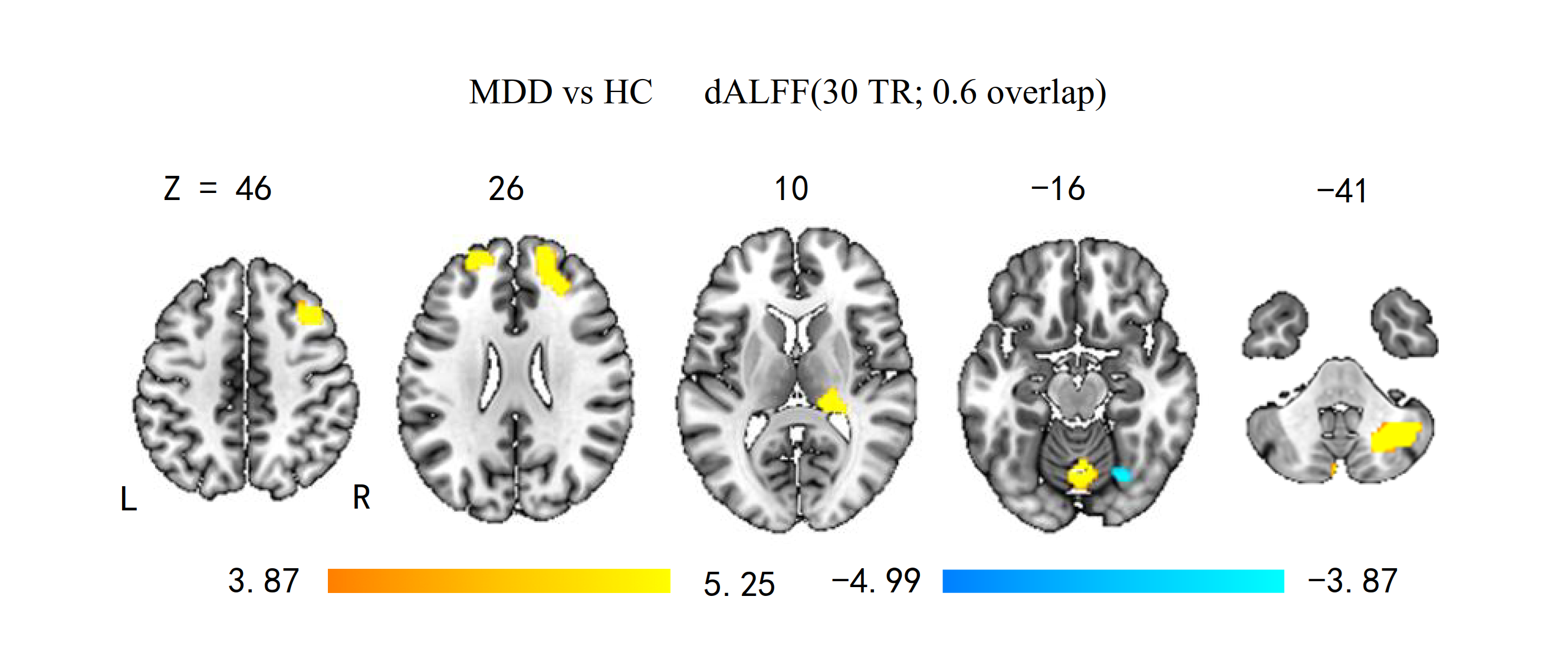

Supplement: Supplementary file 2 [file Image_2.TIF]

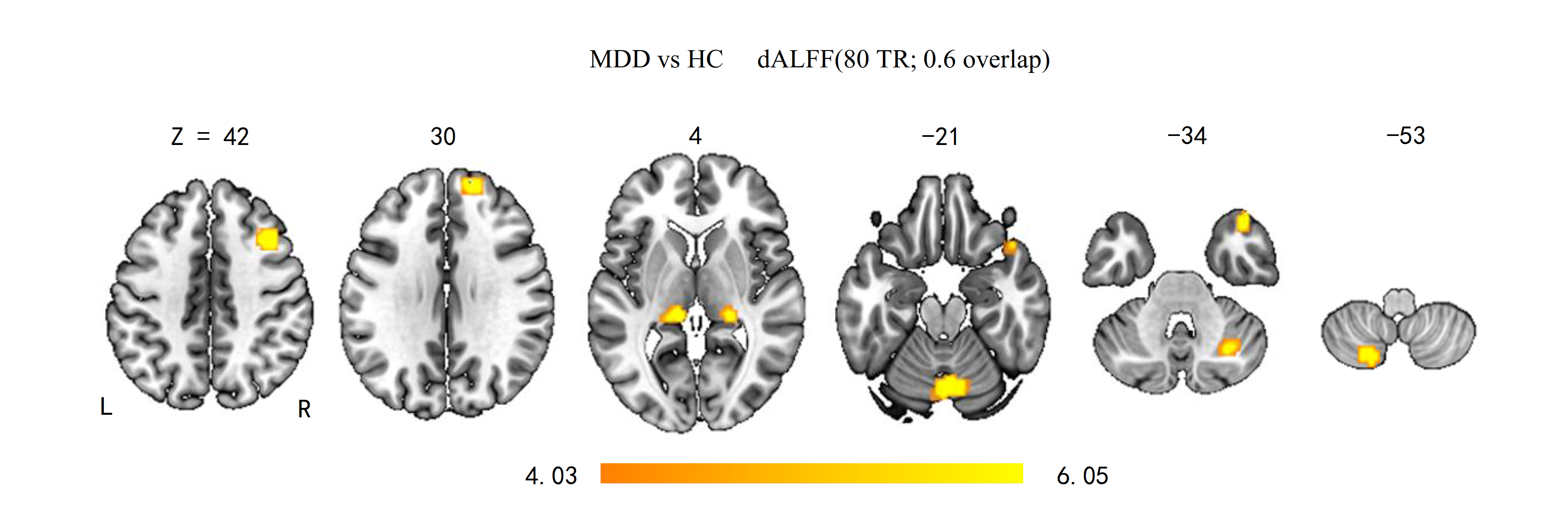

Supplement: Supplementary file 3 [file Image_3.TIF]

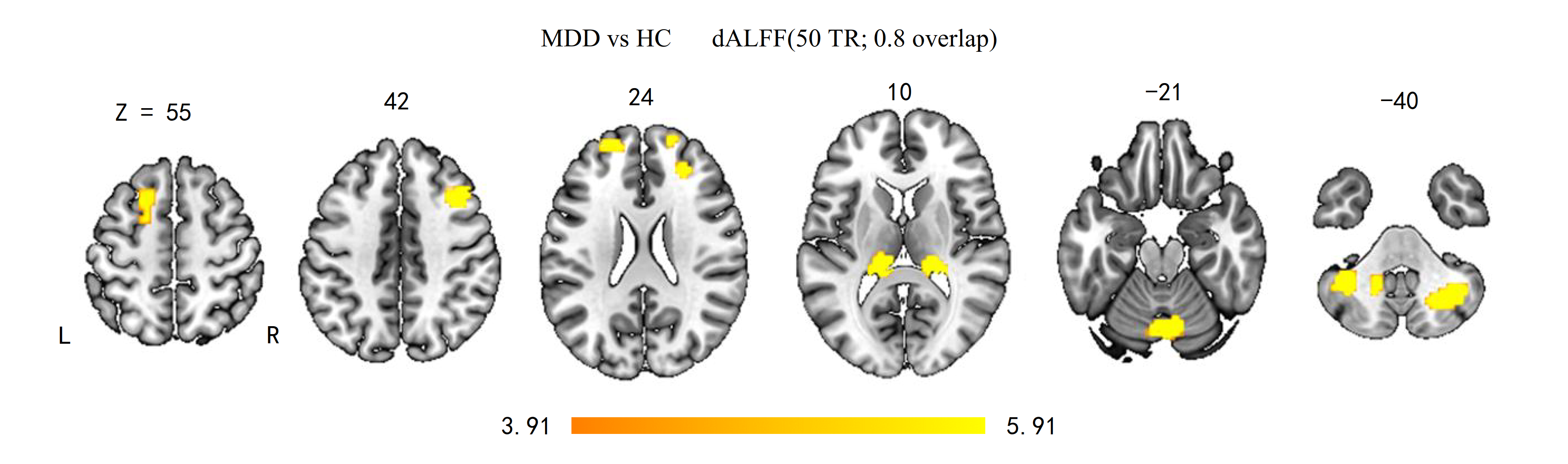

Supplement: Supplementary file 4 [file Image_4.TIF]

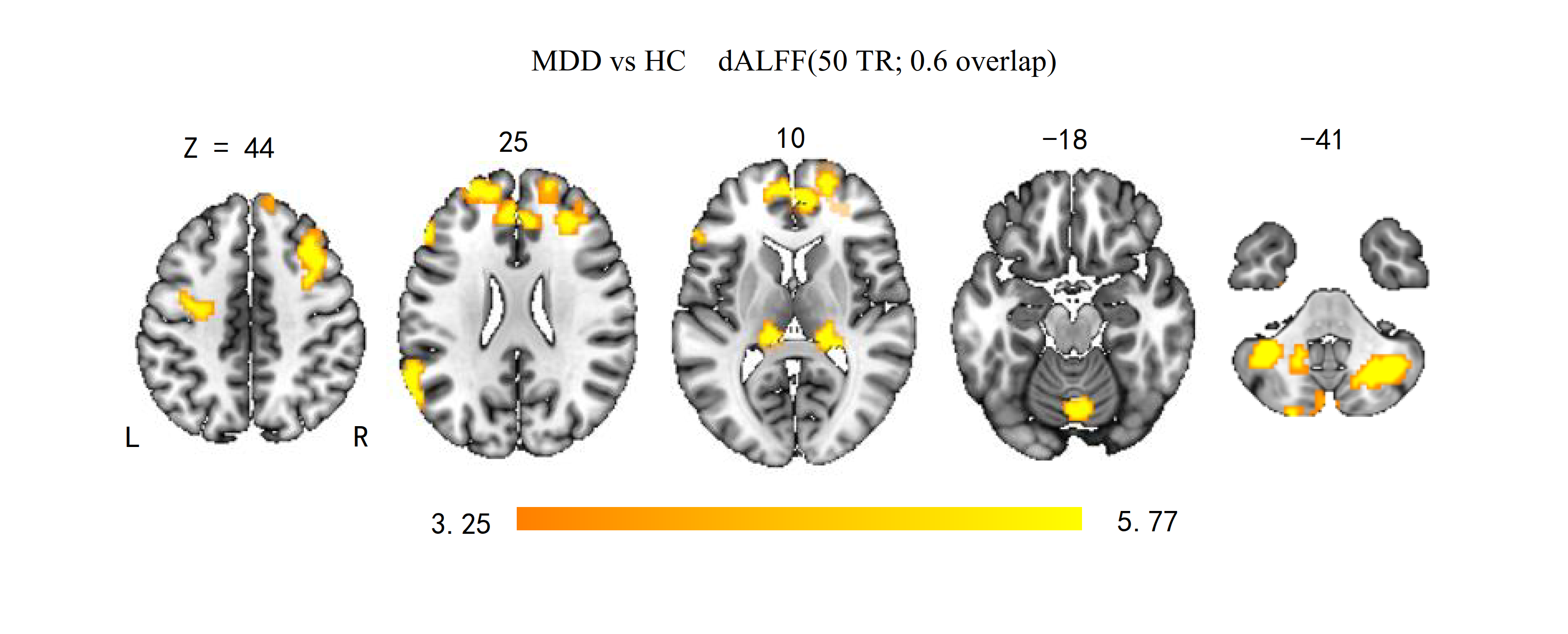

Supplement: Supplementary file 5 [file Image_5.TIF]
