# Supplementary material for: Dynamic Altered Amplitude of Low-Frequency Fluctuations in Patients With Major Depressive Disorder
Source: Front Psychiatry. 2021 Jul 19;12:683610. doi: 10.3389/fpsyt.2021.683610 (PMC8328277; doi:10.3389/fpsyt.2021.683610)
Supplement: Supplementary file 6 [file Data_Sheet_1.docx]

**Supplementarymaterials for**

“Dynamic altered of amplitude of low-frequency fluctuations in patients with major depressive disorder”

Ruiping Zheng^1,3-8#^, PhD, Yuan Chen^1,3-8#^, MD, Yu Jiang^1,3-8^, MD, Mengmeng Wen^1,3-8^, MD, Bingqian Zhou^1,3-8^, MD, Shuying Li^2^, PhD, Yarui Wei^1,3-8^, PhD, Zhengui Yang^1,3-8^, MD, Jingliang Cheng^1,3-8*^, PhD, Yong Zhang^1,3-8*^, PhD, Shaoqiang Han^1,3-8*^, PhD

**Author Affiliation:**

^1^Department of Magnetic Resonance Imaging, the First Affiliated Hospital of Zhengzhou University

^2^Department of Psychiatry, the First Affiliated Hospital of Zhengzhou University

^3^Key Laboratory for functional magnetic resonance imaging and molecular imaging of Henan Province

^4^Engineering Technology Research Center for detection and application of brain function of Henan Province

^5^Engineering Research Center of medical imaging intelligent diagnosis and treatment of Henan Province

^6^Key Laboratory of magnetic resonance and brain function of Henan Province

^7^Key Laboratory of brain function and cognitive magnetic resonance imaging of Zhengzhou

^8^ Key Laboratory of Imaging Intelligence Research medicine of Henan Province

^#^ Ruiping Zheng and Yuan Chen contributed equally to the present work

***Corresponding Author**

Corresponding authors at: Functional and Molecular Imaging Key Laboratory of Henan Province, Department of MRI division, First Affiliated Hospital of Zhengzhou University, Henan, PR China.

E-mail addresses: fccchengjl@zzu.edu.cn (J. Cheng), zzuzhangyong2013@163.com (Y. Zhang), ShaoqiangHan@163.com (S. Han).

**Table S1.**Brain regions showing significant differences insALFF between MDD patientsand HC subjects

**Abbreviations:** sALFF = static amplitude of low-frequency fluctuation; MDD = major depressive disorder; HC = healthy control; MNI = Montreal Neurological Institute; L=left; R=right

| Regions | MNI coordinates | | |  | Voxels | *T* value |
| --- | --- | --- | --- | --- | --- | --- |
|  | *x* | *y* | *z* |  |  |  |
| Calcarine _L | -12 | -54 | 0 |  | 37 | -5.24 |
| Calcarine _R | 18 | -66 | 9 |  | 29 | -4.77 |
| Occipital Lobe _L | -21 | -87 | -18 |  | 58 | -5.64 |
| Cerebellum Posterior Lobe _R | 27 | -72 | -18 |  | 143 | -6.00 |
| Fusiform Gyrus _L | -45 | -75 | -21 |  | 23 | -5.08 |
| Vermis | -3 | -75 | -24 |  | 26 | 4.25 |

**FigureS1.**Brain regions with significant group differences in sALFF. Group differences insALFF between the MDD and HC groups were identified using a two-sample *t*-test. The statistical significance level was set at *q*<0.05, topologicalFDRcorrected. Patients with MDD showed significantlydecreased sALFF in the bilateralcalcarine and left occipital lobe, leftfusiform gyrus and rightCPL and increased sALFF in the vermis.

Abbreviations: sALFF, static amplitude of low-frequency fluctuation; MDD, major depressive disorder; HC, healthy control; CPL, cerebellum posterior lobe.

**FigureS2.**Brain regions with significant group differences in dALFF variability (30 TRs; 0.6 overlap). Group differences of temporal variability of dALFF between the MDD and HC groups were identified using a two-sample *t*-test. The statistical significance level was set at*q*<0.05, topologicalFDRcorrected. Patients with MDD showed significantlyincreased dALFF variability in the bilateral SFG and right MFG, rightthalamus, right CPL and vermis and decreased dALFF variability in the right fusiform gyrus.

Abbreviations: dALFF, dynamic amplitude of low-frequency fluctuation; MDD, major depressive disorder; HC, healthy control; SFG, superior frontal gyrus; MFG, middle frontal gyrus; CPL, cerebellum posterior lobe.

**FigureS3.**Brain regions with significant group differences in dALFF variability (80 TRs; 0.6 overlap). Group differences of temporal variability of dALFF between the MDD and HC groups were identified using a two-sample *t*-test. The statistical significance level was set at*q*<0.05, topological FDRcorrected. Patients with MDD showed significantlyincreased dALFF variability in the bilateralthalamus, bilateral CPLand right MFG, right SFG,right STG, and vermis.

Abbreviations: dALFF, dynamic amplitude of low-frequency fluctuation; MDD, major depressive disorder; HC, healthy control; CPL, cerebellum posterior lobe; MFG, middle frontal gyrus; SFG, superior frontal gyrus; STG, superior temporal gyrus.

**FigureS4.**Brain regions with significant group differences in dALFF variability (50 TRs; 0.8 overlap). Group differences of temporal variability of dALFF between the MDD and HC groups were identified using a two-sample *t*-test. The statistical significance level was set at *q*<0.05, topological FDRcorrected. Patients with MDD showed significantly increased dALFF variability in the bilateral thalamus, bilateral SFG,bilateral CPL and left MFG and vermis.

Abbreviations: dALFF, dynamic amplitude of low-frequency fluctuation; MDD, major depressive disorder; HC, healthy control; SFG, superior frontal gyrus; CPL, cerebellum posterior lobe; MFG, middle frontal gyrus.

**FigureS5.**Brain regions with significant group differences in dALFF variability (50 TRs; 0.6 overlap). Group differences of temporal variability of dALFF between the MDD and HC groups were identified using a two-sample *t*-test. The statistical significance level was set at *q*<0.05, topological FDRcorrected. Patients with MDD showed significantly increased dALFF variability in the bilateral CPL, bilateral SFG, bilateralMFG, bilateral thalamus and left IFG, leftSTG and ACC, vermis.

Abbreviations: dALFF, dynamic amplitude of low-frequency fluctuation; MDD, major depressive disorder; HC, healthy control; CPL, cerebellum posterior lobe; SFG, superior frontal gyrus; MFG, middle frontal gyrus; IFG, inferior frontal gyrus; STG, superior temporal gyrus; ACC, anterior cingulatecortex.
